# Association between dental caries and self-reported COPD prevalence in U.S. adults ages ≥ 60-year: a cross-sectional analysis of NHANES 2015–2020

**DOI:** 10.1080/20002297.2026.2705671

**Published:** 2026-07-21

**Authors:** Chengyu Huang, Wentao Lin, Wen Zhou

**Affiliations:** a Clinical Research Center for Oral Tissue Deficiency Diseases of Fujian Province & Fujian Key Laboratory of Oral Diseases & Fujian Provincial Engineering Research Center of Oral Biomaterial, School and Hospital of Stomatology, Fujian Medical University, Fuzhou, China; b Stomatological Key Laboratory of Fujian College and University & Institute of Oral Homeostasis & Research Center of Oral Tissue Engineering, Fujian Medical University, School and Hospital of Stomatology, Fujian Medical University, Fuzhou, China

**Keywords:** Dental caries, chronic obstructive pulmonary diseases, NHANES, aged, logistic models

## Abstract

**Background:**

Evidence on the association between dental caries and chronic obstructive pulmonary disease (COPD) risk in older adults is limited.

**Objective:**

This cross-sectional study aimed to explore the associations of coronal and root caries with self-reported COPD prevalence in this population.

**Design:**

Data were obtained from the National Health and Nutrition Examination Survey (NHANES) 2015–2020. Coronal caries was measured by the decayed, missing and filled teeth (DMFT) index. Root caries was recorded as presence or absence. Weighted multivariable logistic regression models were applied to assess the associations of coronal caries and root caries with COPD.

**Results:**

A total of 3,972 eligible participants were included, with a weighted self-reported COPD prevalence of 13.68%. Per 1-unit increase in DMFT was associated with higher odds of COPD (OR = 1.04, 95%CI: 1.02–1.06, *p* < 0.001). Participants with root caries had elevated odds (OR = 1.47, 95%CI: 1.02–2.11, *p* = 0.048).

**Conclusions:**

Coronal and root caries are independently associated with higher odds of self-reported COPD in U.S. older adults. However, given the cross-sectional design, causal inference cannot be established, and further prospective research is needed.

## Article highlights

- Each 1-unit in crease in the DMFT index was associated with 4% higher odds of self-reported COPD (OR = 1.04, 95% CI: 1.02–1.06).
- Root caries was associated with 47% higher odds of self-reported COPD (OR = 1.47, 95% CI: 1.02–2.11).
- The associations between dental caries and COPD remain consistent in most subgroups.

## Introduction

Dental caries is a chronic disease caused by an oral microecological imbalance. Its core feature is the abnormal proliferation of acid-producing and acid-tolerant bacteria (e.g*. Streptococcus mutans, Lactobacillus*), which drive the progressive demineralization of dental hard tissues through carbohydrate fermentation [1,2]. Oral microecological imbalance was demonstrated linking to systemic health through pathways such as microbial translocation and systemic inflammatory activation [3]. Dental caries is primarily a localized hard-tissue disease in its early stage. Its potential systemic effects may become more relevant when caries progresses to deep dentin, pulpal involvement, or periapical inflammatory lesions. First, cariogenic pathogens may enter the bloodstream through damaged pulp or periapical tissues. This may lead to transient bacteremia and possible dissemination of microorganisms to distant sites [4]. Second, pro-inflammatory mediators released from local infectious lesions, such as C-reactive protein and interleukin-6, may enter the peripheral circulation and be involved in systemic inflammatory activation [4,5]. Dental caries may contribute to the development or progression of certain systemic diseases, such as ischemic stroke, coronary heart disease and rheumatoid arthritis, through these ways [6,7].

The anatomical continuity between the oral cavity and respiratory tract forms the biological basis of the well-recognized ‘oral–lung axis’: oral microorganisms can translocate to the lower respiratory tract via aspiration or mucosal migration, and their crosstalk with lung microbiota may promote the progression of chronic respiratory diseases (CRDs) [8–10]. Chronic obstructive pulmonary disease (COPD), a leading cause of global mortality and morbidity, is characterized by irreversible airflow limitation and persistent airway inflammation, with tobacco smoking as its most well-established modifiable risk factor [11]. Accumulating evidence has established a robust association between periodontal disease and COPD via the oral–lung axis, which represents the most extensively studied oral-respiratory linkage [12,13]. In comparison, dental caries, which is characterized by distinct microbial dysregulation, has received far less attention in COPD research. Emerging studies have linked a higher caries burden to an increased risk of lower respiratory tract infections, including pneumonia [13–15]. Oral cariogenic bacteria may be inhaled into the respiratory tract, where they may induce host immune responses and aggravate chronic airway inflammation [16,17]. However, direct evidence linking dental caries specifically to COPD remains limited.

According to the criteria established by the World Health Organization (WHO) [18], adults aged 60 years and older are a high-priority population for both dental caries and COPD prevention, with a significantly higher prevalence of both diseases due to age-related physiological changes and cumulative risk factor exposure [19,20]. Nevertheless, epidemiological studies on the association between dental caries and COPD risk in older adults remain scarce, and existing research rarely divides dental caries into coronal and root caries for separate analysis.

To address these critical research gaps, we conducted a population-based study using nationally representative data from the 2015–2020 National Health and Nutrition Examination Survey (NHANES). We aimed to examine the independent associations of coronal and root caries with COPD prevalence in adults aged 60 years and older. As a supplementary exploratory analysis, we tested the mediating effect of root caries on the association between coronal caries and COPD to confirm the independent roles of the two phenotypes. We hypothesized that both coronal caries and root caries are independently associated with higher odds of COPD. Our findings may provide novel population-based evidence for the oral–lung axis hypothesis and inform the development of oral health promotion and COPD primary prevention strategies for older adults.

## Methods

### Study design and participants

Data for this study were obtained from the 2015–2016 NHANES cycle and the NHANES 2017–March 2020 pre-pandemic dataset. NHANES is a nationally representative, ongoing cross-sectional survey conducted by the U.S. Centers for Disease Control and Prevention (CDC). It assesses the health and nutritional status of the U.S. civilian noninstitutionalized population. The 2019–2020 NHANES cycle was suspended in March 2020 because of the COVID-19 pandemic. Therefore, the partial 2019–March 2020 data are not nationally representative as a standalone cycle. To preserve national representativeness, the National Center for Health Statistics (NCHS) combined the partial 2019–March 2020 data with the complete 2017–2018 cycle and released the official 2017–March 2020 pre-pandemic dataset with calibrated survey weights. In the present study, the 2017–March 2020 pre-pandemic dataset was pooled with the complete 2015–2016 cycle. This approach was used to increase the sample size of older adults and improve statistical power. All survey weights, stratification variables, and primary sampling units were applied according to official NCHS guidance for pooled multi-cycle analyzes.

**Figure 1.**
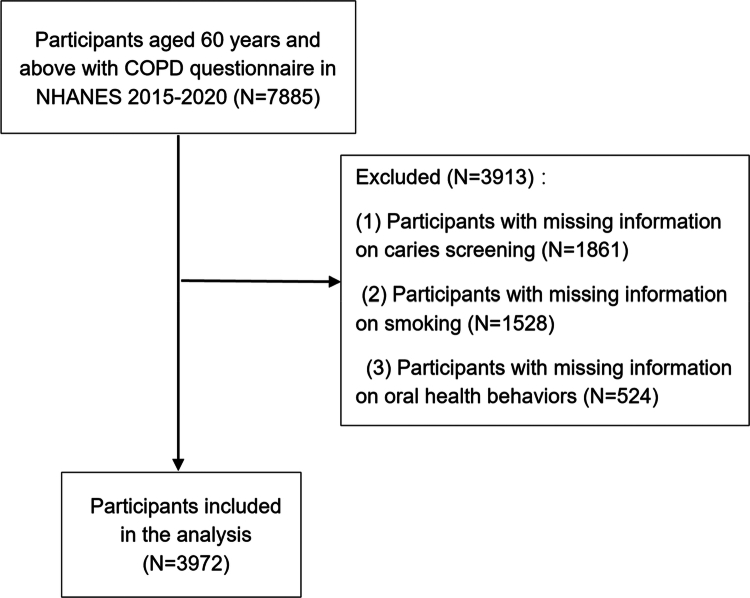
Flowchart for screening participants in this study.

The survey employs a variety of methods to collect data, such as interviews and physical examinations. Interviews gather information on demographics, socioeconomic background, dietary habits and health-related behaviors. Physical examinations include medical assessments, dental examinations and physiological measurements, supplemented by laboratory tests for comprehensive health evaluation. The NHANES study protocol was approved by the Institutional Review Board of the National Center for Health Statistics. All participants provided informed consent for their data to be used in relevant research [21]. Inclusion criteria for this study were: (1) age ≥60 years; (2) completion of dental caries examination and COPD questionnaire. Exclusion criteria were: (1) missing or incomplete data on dental examination or COPD questionnaire; (2) missing smoking data; (3) missing data on oral health behavior questionnaire. A total of 7,885 participants meeting the age and COPD questionnaire requirements were initially identified from the NHANES 2015–2020. After applying the exclusion criteria, 3,913 participants were excluded and 3,972 eligible participants were included in the final analysis. The detailed participant screening process is illustrated in Figure 1.

### Outcomes measures

Chronic bronchitis and emphysema are two clinical manifestations of COPD. Chronic bronchitis is defined as chronic inflammation of the bronchial mucosa, diagnosed by cough and sputum production lasting at least 3 months per year for 2 consecutive years. Emphysema refers to abnormal and persistent dilation of the airspaces distal to the terminal bronchioles, accompanied by destruction of alveolar walls. In clinical practice, these two pathological conditions often coexist, jointly causing the characteristic airflow limitation of COPD [11,22]. Following previous studies, COPD was defined based on self-reported physician diagnosis [23]. It was confirmed using three self-reported questionnaire items: ‘Has a doctor ever told you that you have chronic bronchitis?’, ‘Has a doctor ever told you that you have emphysema?’ and ‘Has a doctor or other health professional ever told you that you have chronic obstructive pulmonary disease?’ Participants who answered ‘yes’ to any of the three questions were classified into the COPD group; those who answered ‘no’ to all three were classified into the non-COPD group [23].

### Dental caries

All oral health examinations in NHANES were performed by trained and calibrated dentists at Mobile Examination Centers (MEC). During the examination, each oral quadrant was dried first. Then, a mouth mirror and probe were used to examine four surfaces (lingual, buccal, mesial, distal) of anterior teeth, and five surfaces (occlusal, lingual, buccal, mesial, distal) of first and second molars [24]. The decayed, missing and filled teeth (DMFT) index was calculated by counting the number of decayed (D), missing (M) and filled (F) teeth [25]. Teeth coded ‘E, F, J, P, R, T, Z’ were used for DMFT calculation [26]. DMFT was treated as a continuous variable to measure coronal caries [27]. Root caries was defined as the presence or absence of root caries detected in the full dentition during examination, with the result recorded as either ‘Yes’ or ‘No’. Participants with complete edentulism (no remaining natural teeth) were excluded from the analysis, as root caries assessment can only be performed among individuals with retained teeth.

### Covariates

Covariates were selected based on prior knowledge, biological plausibility and established epidemiological evidence regarding COPD risk factors, covering demographic characteristics, socioeconomic status, lifestyle factors and oral health behaviors [28,29]. The following variables were included in the fully adjusted model: age (continuous), gender (male/female), race (Mexican American, Non-Hispanic Black, Non-Hispanic White, Other Hispanic, Other Race including Multi-Racial), marital status (Married/Living with partner; Divorced/Separated/Widowed; Never married), education level (Less than high school, High school or equivalent, College or above), poverty-income ratio (PIR, < 1; 1–3; ≥3), body mass index (BMI), smoking status (current, former, never), alcohol consumption (yes/no), last dental visit time and dental floss/device use frequency. Demographic and socioeconomic data were collected via household interviews, and lifestyle and oral health behavior data were obtained from standardized questionnaires.

### Statistical analysis

All statistical analyzes were performed using R version 4.3.0 (R Foundation for Statistical Computing, Vienna, Austria), with a two-sided *p* < 0.05 defined as the threshold for statistical significance. All analyzes accounted for the complex multi-stage probability sampling design of the NHANES 2015–2020 cycles by incorporating the appropriate sample weights, stratification variables and primary sampling units (PSUs) calibrated for the combined survey cycles, to generate unbiased, nationally representative estimates for the U.S. older adult population.

Baseline characteristics of the included older adults were stratified and presented by COPD status. Continuous variables were reported as weighted mean ± standard deviation (SD), and categorical variables were presented as unweighted frequency with weighted percentage [*n* (%)]. Weighted Student's t-tests were used to compare between-group differences in continuous variables, and Rao-Scott adjusted chi-square tests were applied for comparisons of categorical variables.

The associations of coronal caries and root caries with COPD prevalence were evaluated using weighted univariable and multivariable logistic regression models. Results were presented as odds ratios (ORs) with corresponding 95% confidence intervals (CIs). For multivariable analysis, all prespecified confounders were entered into the model simultaneously based on prior knowledge and biological plausibility.

We conducted a mediation analysis to explore the potential role of root caries in the association between coronal caries and COPD prevalence. The mediating effect was considered statistically significant if the following criteria were met: (1) both the total effect and indirect effect were statistically significant and (2) the direction of the indirect effect was consistent with that of the total effect.

In addition, multiplicative interaction tests were performed in fully adjusted weighted logistic regression models to assess the heterogeneity of associations of coronal caries and root caries with COPD across subgroups (gender, age, BMI, education level, marital status, PIR, alcohol consumption and smoking status).

## Results

### Participant characteristics

Table 1 summarizes the baseline characteristics of 3,972 included U.S. adults aged ≥60 years (weighted national population: 58,560,503), stratified by COPD status. The overall weighted COPD prevalence was 13.68% (493 unweighted cases, weighted to 8,009,074 nationally). Participants with COPD had a higher mean DMFT score (17.29 ± 5.88 vs. 15.18 ± 5.96, *p* < 0.001) and higher root caries prevalence (25.1% vs. 13.8%, *p* < 0.001) than the non-COPD group. Other variables with significant between-group differences included education level, PIR, BMI, smoking status and alcohol consumption (all *p* < 0.05).

**Table 1.** Characteristics of the older participants stratified by COPD.

Characteristic	Overall *N* (weighted) = 58,560,503	Non-COPD *N* (weighted) = 50,551,429	COPD *N* (weighted) = 8,009,074	*p-*value
*N* (unweighted)	3,972	3,479	493	
DMFT, Mean ± SD	15.47 ± 5.99	15.18 ± 5.96	17.29 ± 5.88	<.001
**Root caries, *n*(%)**				<.001
No	3,168 (84.6)	2,825 (86.2)	343 (74.9)	
Yes	804 (15.4)	654 (13.8)	150 (25.1)	
**Last dental visit, *n*(%)**				0.095
6 months or less	1,832 (58.8)	1,639 (59.5)	193 (53.9)	
More than 6 months, but not more than 1 year ago	571 (13.4)	497 (13.3)	74 (13.8)	
More than 1 year, but not more than 2 years ago	414 (7.8)	359 (8.0)	55 (7.1)	
More than 2 years, but not more than 3 years ago	292 (5.5)	258 (5.6)	34 (5.2)	
More than 3 years, but not more than 5 years ago	274 (5.1)	237 (4.7)	37 (7.2)	
More than 5 years ago	546 (8.9)	449 (8.4)	97 (12.5)	
Never have been	43 (0.5)	40 (0.6)	3 (0.3)	
Dental floss/device use, Mean ± SD	3.99 ± 2.96	3.99 ± 2.95	3.96 ± 3.01	0.883
Age, Mean ± SD	69.37 ± 6.62	69.37 ± 6.60	69.41 ± 6.76	0.951
**Gender, *n*(%)**				0.93
Female	1,970 (54.1)	1,726 (54.1)	244 (53.8)	
Male	2,002 (45.9)	1,753 (45.9)	249 (46.2)	
**Race, *n*(%)**				0.089
Mexican American	484 (4.1)	451 (4.3)	33 (2.6)	
Non-Hispanic Black	467 (4.5)	425 (4.7)	42 (3.2)	
Non-Hispanic White	1,628 (75.4)	1,367 (74.8)	261 (79.0)	
Other Hispanic	929 (8.4)	808 (8.5)	121 (8.3)	
Other Race – Including multi-racial	464 (7.6)	428 (7.7)	36 (6.9)	
**Marital status, *n*(%)**				0.086
Married/living with partner	2,319 (64.3)	2,079 (65.3)	240 (57.3)	
Divorced/separated/widowed	1,416 (31.4)	1,202 (30.7)	214 (35.9)	
Never married	237 (4.4)	198 (4.0)	39 (6.7)	
**Education Level, *n*(%)**				0.002
Less than high school	468 (4.6)	424 (4.8)	44 (3.5)	
High school or equivalent	1,352 (32.3)	1,143 (30.6)	209 (42.9)	
College or above	2,152 (63.1)	1,912 (64.6)	240 (53.6)	
BMI, Mean ± SD	29.55 ± 6.44	29.27 ± 6.12	31.32 ± 7.95	<0.001
**Smoking status, *n*(%)**				<0.001
Current	472 (9.4)	358 (7.7)	114 (19.9)	
Former	1,390 (37.4)	1,156 (35.5)	234 (49.3)	
Never	2,110 (53.2)	1,965 (56.8)	145 (30.8)	
**PIR, *n*(%)**				<.001
<1	360 (14.30)	309 (14.10)	51 (15.64)	
1–3	1,135 (45.08)	950 (43.34)	185 (56.75)	
≥3	1023 (40.63)	933 (42.56)	90 (27.61)	
**Alcohol consumption, *n* (%)**				0.012
No	1,979 (44.6)	1,732 (43.2)	247 (53.3)	
Yes	1,993 (55.4)	1,747 (56.8)	246 (46.7)	

### Associations between dental caries and COPD

Table 2 presents the associations of coronal caries and root caries with COPD prevalence in older adults, derived from three progressively adjusted weighted logistic regression models. In the unadjusted crude model (Model 1) and the multivariable model adjusted for all prespecified confounders based on prior knowledge (Model 2), both higher DMFT and the presence of root caries were significantly associated with higher odds of COPD (all *p* < 0.05).

**Table 2.** Weighted logistic regression results for the associations of caries and COPD in older adults.

Analysis	Variables	OR (95%CI)	*p-*value
Model 1	DMFT	1.06 (1.04–1.08)	<0.001
	Root caries		
	No	1.00 (Reference)	
	Yes	2.09 (1.42–3.06)	<0.001
Model 2	DMFT	1.04 (1.02–1.06)	<0.001
	Root caries		
	No	1.00 (Reference)	
	Yes	1.61 (1.14–2.27)	0.011
Model 3	DMFT	1.04 (1.02–1.06)	<0.001
	Root caries		
	No	1.00 (Reference)	
	Yes	1.47 (1.02–2.11)	0.048

OR: Odds Ratio, CI: Confidence Interval Model 1: Unadjusted crude model. Model 2: Multivariable model adjusted for all prespecified confounders based on prior knowledge: age, gender, race, education level, marital status, BMI, PIR, smoking status, alcohol consumption, last dental visit and dental floss device. Model 3: Fully adjusted model, adjusted for all confounders included in Model 2, with mutual adjustment for DMFT and root caries.

To further verify the independent associations of DMFT and root caries with COPD, both variables were simultaneously incorporated into the fully adjusted model (Model 3) for mutual adjustment. After adjustment, higher DMFT remained significantly associated with higher odds of COPD (OR = 1.04, 95%CI: 1.02–1.06, *p* < 0.001), with each 1-unit increase in DMFT corresponding to 4% higher odds of COPD. The presence of root caries also retained a significant positive association with COPD: compared with the reference group (no root caries), older adults with root caries had 47% higher odds of COPD (OR = 1.47, 95%CI: 1.02–2.11, *p* = 0.048).

### Mediating role of root caries

Weighted mediation analysis was conducted (adjusted for all confounding factors included in the final multivariable model) to assess the mediating effect of root caries on the association between coronal caries and COPD, with results presented in Table 3.

**Table 3.** Mediation effect of root caries in associations of coronal caries with COPD in older adults.

Effect	*β* (95%CI)	*p-*value	Proportion mediated
Indirect	0.0002 (−0.0006–0.0010)	0.674	4.73
Direct	0.0024 (0.0015–0.0031)	<0.001	95.27
Total	0.0026 (0.0014–0.0036)	<0.001	100.00

The results showed that root caries was not a statistically significant mediator in the association between coronal caries and COPD, which did not meet the pre-specified significance criteria for mediation effect (indirect effect *β* = 0.0002, 95%CI: −0.0006–0.0010, *p* = 0.674). The association between coronal caries and COPD was dominated by the direct effect of coronal caries (*β* = 0.0024, 95%CI: 0.0015–0.0031, *p* < 0.001), which accounted for 95.27% of the total effect.

### Stratified analysis

**Figure 2.**
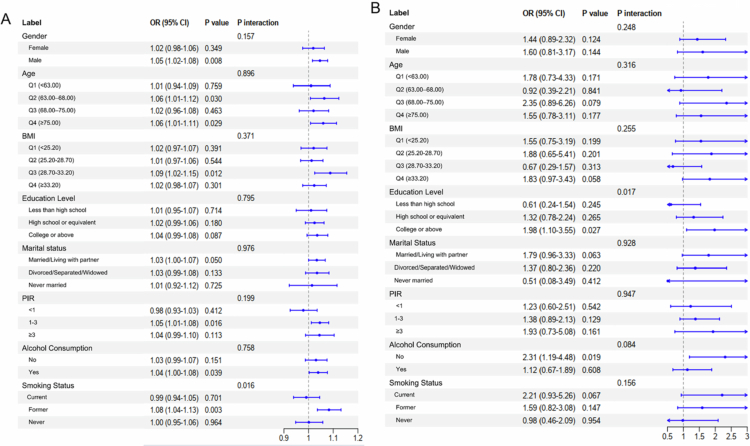
Subgroup analyzes of the associations of coronal and root caries with COPD in older adults. A. Subgroup analysis of the association between coronal caries experience and COPD in the older adults. B. Subgroup analysis of the association between root caries and COPD in the older adults. All analyzes were performed using weighted multivariable logistic regression models, adjusted for: (A) age, gender, race, education level, marital status, BMI, PIR, smoking status, alcohol consumption, last dental visit, dental floss device and root caries; (B) age, gender, race, education level, marital status, BMI, PIR, smoking status, alcohol consumption, last dental visit, dental floss device and DMFT.

Figure 2 presents subgroup analyzes evaluating the associations of coronal and root caries with COPD in older adults. In weighted multivariable-adjusted logistic regression models, the significant positive associations of coronal caries and root caries with COPD remained consistent across most subgroups. Interaction tests revealed that the association between coronal caries (DMFT) and COPD was significantly modified by smoking status (*p* for interaction = 0.016). Stratified analyzes showed that the positive association between coronal caries and COPD was more pronounced in older adults who were former smokers (OR = 1.08, 95%CI: 1.04–1.13). Meanwhile, the association between root caries and COPD was significantly modified by education level (*p* for interaction = 0.017), with the most prominent positive association observed in participants with a college education or above (OR = 1.98, 95%CI: 1.10–3.55). No significant effect modification was detected in other subgroups, indicating that the associations of coronal caries and root caries with COPD were stable and homogeneous across these strata.

## Discussion

Accumulating epidemiological evidence has linked poor oral health to chronic respiratory diseases [30–32]. Emerging studies demonstrated a higher caries burden related to an increased risk of lower respiratory tract infections, including pneumonia [13–15]. However, dental caries has received less attention in COPD research. In the present nationally representative sample of older adults, both coronal caries and root caries were associated with higher odds of COPD. In the fully adjusted model, each 1-unit increase in the DMFT index was associated with 4% higher odds of COPD (OR = 1.04, 95%CI: 1.02–1.06, *p* < 0.001). Participants with root caries had 47% higher odds of COPD than those without root caries (OR = 1.47, 95%CI: 1.02–2.11, *p* = 0.048). These findings are consistent with the literature linking oral disease burden to respiratory health [12]. For example, previous NHANES-based studies have reported an association between tooth loss and COPD [33]. However, tooth loss is a broad oral health endpoint and cannot distinguish the potential roles of dental caries and periodontal disease. Our study extends this evidence by focusing specifically on dental caries and examining coronal and root caries separately.

Age-related changes, including reduced salivary function, gingival recession with root surface exposure and denture use, may increase susceptibility to dental caries, especially root caries [34]. COPD prevalence also increases with age, and age-related decline in lung function may increase vulnerability to harmful exposures [35,36]. These shared age-related factors may partly explain why the association between dental caries and self-reported COPD is important in older adults.

Oral pathogens associated with coronal caries may reach the lower respiratory tract via micro-aspiration, contributing to airway infection and inflammation [16]. Meanwhile, pulpitis and periapical periodontitis caused by caries may activate systemic inflammatory responses via the bloodstream, elevating levels of inflammatory factors like C-reactive protein and interleukins [5]. These factors have been proposed to accelerate airway remodeling and disease progression in COPD [5,37,38]. For root caries, the distinct root surface plaque microbiome, characterized by cross-kingdom interactions between *Candida albicans* and cariogenic bacteria, may play a unique role [39,40]. Clinical studies have reported high *Candida* isolation rates in lower respiratory tract samples from patients with acute exacerbations of COPD [41]. *C. albicans* colonization has been shown to amplify pulmonary inflammation via macrophage-mediated pathways [42–44]. It is therefore postulated that root caries-associated *C. albicans* may migrate to the lower respiratory tract through oral secretions and contribute to COPD pathogenesis, though direct evidence is still lacking.

Subgroup analyzes showed that the positive associations of coronal caries and root caries with COPD remained stable across most stratified populations. Interaction tests identified significant effect modification by education level on the association between root caries and COPD (*p* for interaction = 0.017), with the significant positive association only observed in older adults with college education or above, which may be related to differences in health literacy and oral health care utilization across education levels [45,46]. Meanwhile, smoking status significantly modified the association between coronal caries and COPD (*p* for interaction = 0.016), with the most prominent association detected in former smokers, potentially due to the cumulative effect of smoking on both oral and respiratory systems [33,47]. These results further verified the robustness of the associations between coronal caries, root caries and COPD prevalence.

Periodontal disease is a well-established oral condition associated with COPD through the oral–lung axis. This association may involve aspiration or mucosal migration of oral pathogens, as well as systemic inflammatory activation [30]. In older adults, periodontal disease and dental caries frequently coexist. They also share several common risk factors, such as poor oral hygiene and smoking [48]. Therefore, potential confounding by periodontal disease should be carefully considered. In the present study, standardized full-mouth periodontal examination data were not available across the included NHANES 2015–2020 cycles. Therefore, periodontal status could not be directly adjusted in the regression models. Residual confounding by periodontal disease cannot be excluded. The observed associations between caries status and self-reported COPD remained significant after adjustment for a broad set of available confounders, including smoking status, dental visit history and dental floss/device use. These findings should therefore be interpreted cautiously. They do not rule out the influence of periodontal disease. Instead, they suggest that dental caries may provide additional information on the oral health burden in relation to COPD prevalence. Future studies with data on both periodontal disease and dental caries are needed to clarify their separate and combined associations with COPD.

This study found that coronal and root caries were each associated with higher odds of self-reported COPD in older adults. It provides a new research perspective and theoretical basis for the collaborative prevention and control of oral health and respiratory diseases in older adults. However, this study has several limitations. First, the cross-sectional design precludes definitive causal inference between dental caries and COPD; future prospective cohort studies are needed to establish temporal relationships. Second, COPD was defined by self-reported responses to three questionnaire items, which may introduce recall bias, information bias and particularly misclassification bias. This may also include participants with conditions such as chronic bronchitis who do not meet full COPD diagnostic criteria, potentially affecting case ascertainment accuracy. Third, the analysis was limited to pre-pandemic NHANES data through March 2020 because of the COVID-19-related interruption. Therefore, caution is needed when generalizing these findings to post-pandemic populations.

## Conclusions

This nationally representative cross-sectional study demonstrates that both coronal caries and root caries are independently associated with higher odds of self-reported COPD in U.S. older adults ages ≥60-year. Future prospective cohort studies are warranted to clarify the temporal and potential causal relationships between different caries phenotypes and COPD.

## Data Availability

The data presented in this study are available in the NHANES database at https://wwwn.cdc.gov/nchs/nhanes/.
